# How do root fungi of *Alnus nepalensis* and *Schima wallichii* recover during succession of abandoned land?

**DOI:** 10.1007/s00572-023-01124-6

**Published:** 2023-09-13

**Authors:** Sujan Balami, Martina Vašutová, Vijay Kumar Chaudhary, Pavel Cudlín

**Affiliations:** 1 Department of Botany, Faculty of Science, University of South Bohemia, Na Zlaté stoce 1, Ceské Budejovice, 37005 Czech Republic; 2 CAS Key Laboratory of Tropical Forest Ecology, Xishuangbanna Tropical Botanical Garden, Chinese Academy of Sciences, Menglun, Yunnan 666303 China; 3 Central Department of Botany, Tribhuvan University, Kirtipur, Kathmandu, 44600 Nepal; 4 Department of Ecosystem Function Analysis of the Landscape, Czech Academy of Sciences, Lipová 1789/9, Ceské Budejovice, 37005 Czech Republic

**Keywords:** Arbuscular mycorrhiza, Ectomycorrhiza, ECM morphotype, Short-term and long-term abandoned soil, Nepal

## Abstract

*Alnus nepalensis* and *Schima wallichii* are native tree species accompanying succession in abandoned agricultural land in the middle mountainous region of central Nepal. To understand how root fungi recover during spontaneous succession, we analyzed the diversity and composition of arbuscular mycorrhizal (AM), ectomycorrhizal (ECM), and total fungi in tree fine roots from three land use types, short-term abandoned land (SA), long-term abandoned land (LA), and regenerated forest (RF) as a reference. Additionally, ECM morphotypes were examined. The results showed different speeds of succession in the studied fungal groups. While the change in the AM fungal community appears to be rapid and LA resembles the composition of RF, the total fungi in the abandoned land types are similar to each other but differed significantly from RF. Interestingly, the relative abundance of Archaeosporaceae followed a trend differing between the tree species (SA < LA in *A. nepalensis*, but SA > LA in *S. wallichii*). Unlike AM and total fungi, there was no significant difference in the ECM community of *A. nepalensis* between land use types, probably due to their low species diversity (9 ECM morphotypes, 31 ECM operational taxonomic units). However, *Cortinarius* sp. was significantly more abundant in RF than in the other land use types, whereas *Alnicola*, *Tomentella*, and *Russula* preferred young stages. Our results suggest that for both studied tree species the AM fungal succession could reach the stage of regenerated forest relatively fast. In the case of total fungi, because of hyperdiversity and composed of species specialized to a variety of environments and substrates, the transition was expected to be delayed in abandoned land where the vegetation was still developing and the ecosystem was not as complex as that found in mature forests.

**Supplementary Information:**

The online version contains supplementary material available at 10.1007/s00572-023-01124-6.

## Introduction

Abandonment of agricultural land is one of the common forms of land use change worldwide caused by socioeconomic and environmental changes (Prishchepov et al. 2012; Estel et al. 2015; Li et al. 2018). It can be defined as the discontinuation of agricultural activities and the complete withdrawal of agricultural management on land (Anguiano et al. 2008). Such abandoned areas have provided opportunities to restore seminatural ecosystems and their functions (Bowen et al. 2007; Yang et al. 2019; Bell et al. 2021). During spontaneous secondary succession in abandoned areas, the entire system including microclimate conditions, soil properties, vegetation, fauna, and associated microbial communities changes continuously (Prévosto et al. 2011; Hannula et al. 2017; Zhang et al. 2017). However, this process is influenced by both local conditions and propagule availability and often will not return to its original state (Cava et al. 2018; Rozendaal et al. 2019).

Abandonment of agricultural land is common in the middle mountainous region of Nepal due to geographical adversity and socioeconomic changes (Chaudhary et al. 2020). After abandonment of fields used for potato, corn, and millet cultivation, these areas are colonized by grasses, sparse shrubs, and tree species such as *Alnus nepalensis* and *Schima wallichii*. Later, the grasses are outcompeted by an increase in the density of shrubs and *A. nepalensis* and *S. wallichii* along with other trees, e.g., *Symplocos ramosissima*, *Daphniphyllum himalayense*, and *Prunus cerasoides*. Both *A. nepalensis* and *S. wallichii* are also a component of seminatural regenerated forests dominated by *D. himalayense*, *Symplocos* spp., *Rhododendron arboreum*, etc. In our previous study (Balami et al. 2021), we observed a significant effect of land use (agricultural land, abandoned land, and regenerated forests) on soil fungal communities. Fungal succession was found to be non-straightforward, as vegetation and total fungal communities appear to be hindered by soil properties and absence of host trees. To disentangle the complex processes in plant and fungal communities during spontaneous succession of abandoned agricultural land, we focused on the root fungi of two tree species, *A. nepalensis* and *S. wallichii*, accompanying succession of such abandoned areas. These trees are of high economic importance to the local community (firewood, animal fodder, timber), play an important role in landslide control, and are able to grow in areas invaded by the non-native *Ageratina adenophora*.

*Alnus nepalensis*, similar to other *Alnus* species, belongs to nitrogen-fixing plants that have a symbiotic relationship with actinobacteria of the genus *Frankia* (Benson and Clawson 2000). Nitrogen fixation is highly demanding for phosphorus, which in the case of *Alnus* is provided by two different groups of fungal symbionts—arbuscular mycorrhizal (AM) and ectomycorrhizal (ECM) fungi. ECM fungi associated with *Alnus* are considered to be more important in nutrient uptake and are dominant in mature forests and under wet conditions (Tedersoo et al. 2009). AM fungi associated with *Alnus* are probably more adapted to warmer and drier conditions (Kilpeläinen et al. 2020). Both mycorrhizal types were found to be structured by climatic and spatial variables (Põlme et al. 2013, 2016). Furthermore, neighboring trees and the local salinity have been found to affect *Alnus* symbionts (Bogar and Kennedy 2013; Thiem et al. 2018).

The symbiotic relationship of *Schima wallichii* is less clear. AM fungi such as *Dentiscutata erythropus*, *Funneliformis geosporum*, *Glomus magnicaule*, *Racocetra gregaria*, *Sclerocystis taiwanensis*, and *Scutellospora* sp. have been reported by Pandey et al. (2016) in *S. wallichii*. However, some reports on ECM fungi have also been published (Ajungla and Jamir 2006), and fruitbodies of ECM species were often found in forests with *S. wallichii* (pers. observ.).

The importance of root-associated fungal communities in restoration processes was highlighted by Neuenkamp et al. (2019), who reported that the addition of mycorrhizal fungi to restoration sites can facilitate the establishment of vegetation cover and encourage the restoration of diverse plant communities that are more similar to reference forests. It is generally known that mycorrhizal fungi are crucial for nutrient acquisition and increase the tolerance to abiotic stress and resistance to pathogens (Smith and Read 2008). However, the whole community of root-associated fungi (total fungi) can also affect tree fitness; for example, root-associated endophytic fungi can enhance resistance to both pathogens and herbivorous insects (Yan et al. 2019; Mehta et al. 2019).

We hypothesized that after abandonment, the diversity of mycorrhizal (AM and ECM) fungi and total fungi associated with *A. nepalensis* and *S. wallichii* would increase due to a subsequent reduction in disturbance during succession and a low disturbance level in regenerated forests (i). The community composition on abandoned land will be different from that of regenerated forests (used as a reference) due to differences in the tolerance of individual fungal species to disturbance, availability of propagules, and the effect of neighboring plants (ii). Due to different levels of host/substrate specificity, the host tree will be more important than land use for total fungi, but not for AM fungi (iii).

## Materials and methods

### Study sites and land use types

**Fig. 1:**
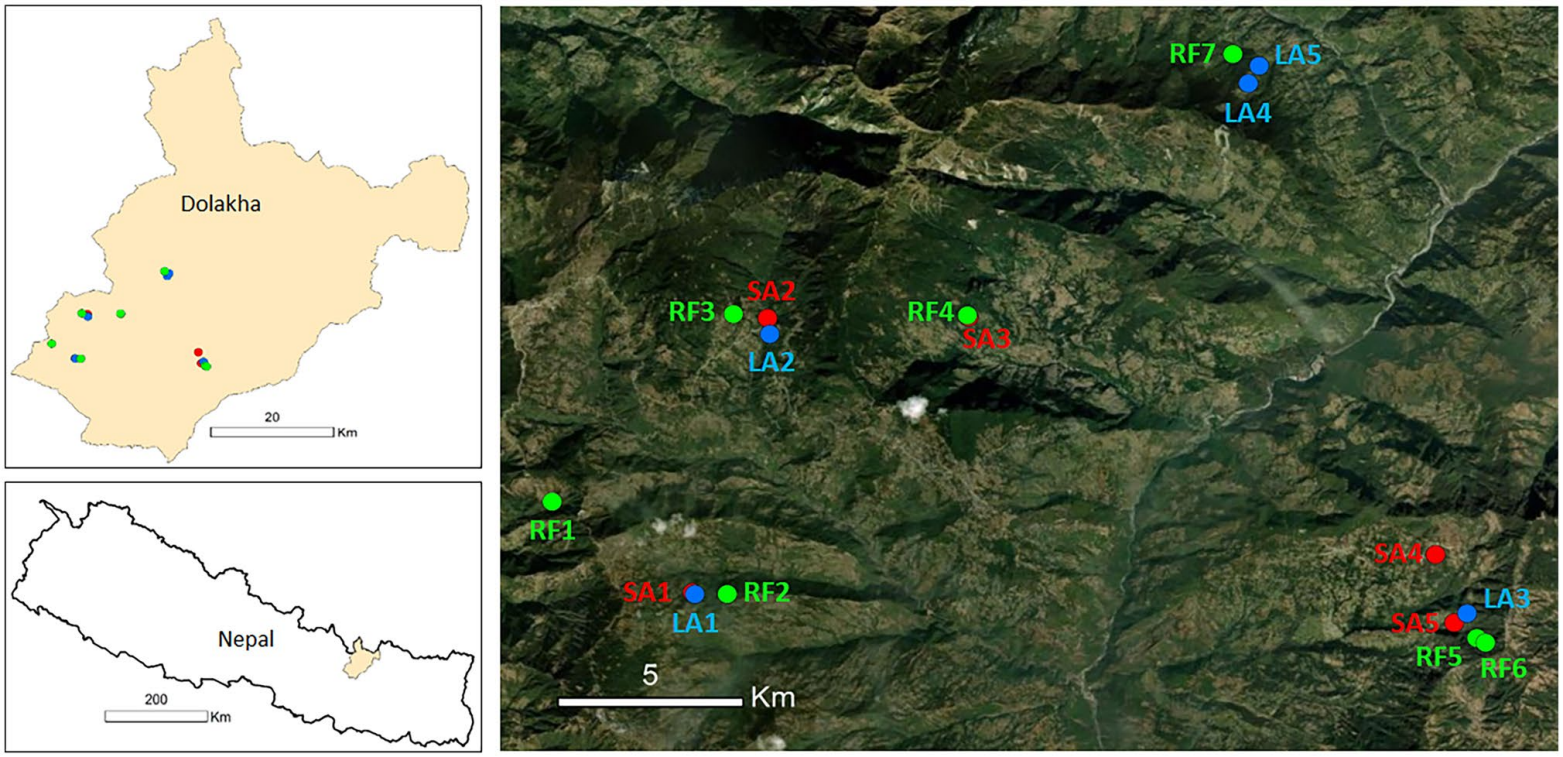
Map of the study area showing sampling sites (SA short-term abandoned land, LA long-term abandoned land, RF regenerated forest)

The study was carried out in Dolakha District, Bagmati Province, central Nepal (Fig. 1; Table S1). The climate in the study area is characterized by hot and humid summers (including a monsoon) and dry and cold winters. The vegetation of the study sites corresponds to the upper belt of subtropical forests and consists mostly of temperate forests with common tree species such as *A. nepalensis*, *S. wallichii*, *Daphniphyllum himalayense*, *Rhododendron arboreum*, and *Symplocos ramosissima*. The soil types are mainly regosols and cambisols. Based on our previous study (Balami et al. 2021), three different types of land use were selected in the study area, namely, two successional stages of abandoned land, short-term abandoned (SA) and long-term abandoned (LA) land, and regenerated forests (RF) as a reference (Table 1). The long-term and short-term regenerated forests in Balami et al. (2021) were considered as regenerated forests (RF) in this study. There are no pristine forests in the studied area due to the long-term exploitation of the landscape by rural populations dependent on local forest resources. Trees were sampled within plots established by Balami et al. (2021) or up to a distance of 30 m from the plots. Descriptions of vegetation and soil properties of plots are available in Balami et al. (2021).

**Table 1:** Land use types and their land use history

**Land use types**	**Land use history**
Short-term abandoned land (SA)	Abandoned for 4–6 years, dominated by herbs and sparse shrubs, without pasture
Long-term abandoned land (LA)	Abandoned for 15–20 years, colonized with younger trees and shrubs, without pasture
Regenerated forest (RF)	Regenerated 30–40 years after disturbance such as pasture, cutting and logging, and litter removal or near natural forests with no known history of forest clearing but affected by disturbance like pasture and litter removal

### Tree species

*Alnus nepalensis* (family: Betulaceae) is a medium to large-sized deciduous tree with its distribution range from 500 to 2600 m asl along the Himalaya (India, Nepal, Bhutan, Tibet, Myanmar, east and west China). It is a pioneer tree that readily colonizes landslide-affected areas, abandoned land, etc. and is also common in forest areas (Sharma and Ambasht 1984). *Schima wallichii* (family: Theaceae) is a medium to large-sized evergreen tree with its distribution range from 900 to 2100 m asl along the Himalaya (Nepal, Bhutan, northeast India, east and west China). This tree also grows in abandoned land and forest areas (Hauchhum and Singson 2020).

### Fine roots sampling

All sampled trees were of relatively similar height (ca. 6–8 m) and diameter at breast height (dbh, ca. 20 cm). The identity of *A. nepalensis* roots was confirmed based on the presence of *Frankia* root nodules and *S. wallichii* roots based on the characteristic pinkish bark and soft texture. Altogether 45 trees (15 trees/land use type) of *A. nepalensis* and 30 trees (10 trees/land use type) of *S. wallichii* (due to their low abundance in SA) were sampled from three land use types SA, LA, and RF. From each tree, three lateral roots (30–40 cm long) were excavated and collected in zip bags. The roots were then transported to the field station and immediately processed (washing followed by fine root sorting). Fine root (dia. ≤ 1 mm) samples were collected in two forms: (i) ECM root tips and (ii) composite root samples.

First, the ECM morphotypes were sorted from each tree sample according to their external morphology. Photographs of the ECM morphotypes were taken using a digital stereomicroscope (Dino-Lite). Two samples per each ECM morphotype (155 in total) were placed separately in a 1.5-ml Eppendorf tube with ~ 10 silica gel beads. No ECM morphotype was found on the roots of *S. wallichii*. We decided to use morphotyping and Sanger sequencing of identified ECM morphotypes because it is a useful tool to verify the trophic status of potentially ECM species in areas with low knowledge on mycobiota. The remaining fine roots of each tree were then cut into small pieces with sterile scissors and homogenized to obtain a composite root sample. The composite root samples were immediately dried in silica gel for further molecular analysis.

### Molecular analyses

DNA from selected ECM morphotypes was extracted using the DNeasy^®^ Plant Mini Kit (Qiagen) according to the manufacturer’s recommendation. Template DNA was amplified using the ITS1F and ITS4 primers. Each 10 µl of PCR reaction mixture contained 0.4 µl of template DNA, 5.0 µl Plain PPMix, 0.6 µl of each primer (5 pmol/µl), and 3.4 µl of sterile water. The PCR conditions were set at a denaturation temperature of 94 °C for 4 min, followed by 35 cycles of 94 °C for 1 min, 56 °C for 30 s, 72 °C for 1 min, and final elongation at 72 °C for 2 min 30 s. After cleaning with ExoAP, the amplicons were sequenced (Sanger sequencing) at Eurofins Genomics (Germany). Only 15 out of 38 ECM morphotype samples were successfully sequenced.

DNA from the composite root samples was extracted from 2 × 30 mg of each root sample (in duplicate) using a Power Soil Kit (Qiagen) according to the manufacturer’s recommendation. The duplicate DNA samples were then pooled into a single sample and then the amplification of the template DNA was performed in triplicate.

The SSU rDNA fragment of AM fungi was amplified by semi-nested PCR. In the first PCR, each 10 µl of the PCR reaction mixture contained 0.5 µl of template DNA, 0.1 µl (2 U/µl) of the polymerase (Phusion High-Fidelity DNA polymerase, New England Biolabs), 2 µl of 5 × HF buffer, 0.2 µl of dNTP (10 mM each dNTP), 0.8 µl of each primer NS31 (Simon et al. 1992) and AML2 (Lee et al. 2008), and 5.6 µl of sterile water. The PCR conditions were set at 94 °C for 3 min, then 35 cycles were run at 94 °C for 30 s, 65 °C for 30 s, 72 °C for 1 min, and the final elongation was run at 72 °C for 10 min. The reaction mixture (10 µl) for the second PCR contained 0.5 µl of template DNA, 0.1 µl (2 U/µl) of the polymerase (Phusion High-Fidelity DNA polymerase, New England Biolabs), 2 µl of 5 × HF buffer, 0.2 µl of dNTP (10 mM of each dNTP), 0.8 µl of each barcoded primer (WANDA (Dumbrell et al. 2011) and AML2), and 5.6 µl of sterile water. The PCR conditions were set at 94 °C for 3 min, then 10 cycles were run at 94 °C for 30 s, 60 °C for 30 s, 72 °C for 1 min, and the final elongation was run at 72 °C for 10 min.

The ITS2 region of total fungi (including ECM fungi) was amplified using the barcoded primers gITS7 and ITS4 (Ihrmark et al. 2012). Each 10 µl of PCR reaction mixture contained 0.5 µl of template DNA, 0.1 µl (2 U/µl) of polymerase (Phusion High-Fidelity DNA polymerase, New England Biolabs), 2 µl of 5 × HF buffer, 0.2 µl of dNTP (10 mM each dNTP), 1 µl of each primer gITS7 and ITS4, and 5.2 µl of sterile water. The PCR conditions were set at a denaturation temperature of 98 °C for 1.5 min, followed by 40 cycles of 98 °C for 1 min, 66 °C for 30 s, 72 °C for 45 s, and final elongation at 72 °C for 10 min.

Amplicons were pooled and purified using a MinElute PCR purification kit (Qiagen) according to the manufacturer’s recommendation. The concentration of PCR products was measured using the Qubit^®^ 2.0 Fluorometer (Thermo Scientific). Sequencing of the amplicons was performed on an Illumina MiSeq (250 bp paired-end) at the SeqMe Company (Czech Republic).

### Bioinformatics

The sequence data of ECM root tips were edited using FinchTV (v1.4.0) and then blasted against the NCBI database. The identification of the ECM morphotype was based on the following criteria: at the species level, sequence similarity ≥ 97% and at the genus level, sequence similarity ≥ 80%. The ECM morphotype sequences were submitted to GenBank with accession numbers ON870288–93, ON908896–9, and OP136002 (Table S6).

The AM amplicon sequencing data (3,240,093 reads) of composite root samples from Illumina MiSeq were processed using the pipeline SEED (v2.1) (Větrovský and Baldrian 2013). For AM fungi, because of the length of the amplified SSU region, only sequences starting from the forward primer were used. Chimeric sequences were removed using the USEARCH (v8.1.1861) UCHIME algorithm (Edgar et al. 2011), and sequences with an expected error rate greater than 0.005 were removed. After resampling (7000 sequences/sample), the dataset was subjected to a BLAST + (v2.5.0) search against the MaarjAM database (accessed in February 2022) using the SSU pipeline (Vasar et al. 2017). The following criteria were required for a match: sequence similarity ≥ 97%; alignment length not differing from the length of the shorter query and subject sequences by > 5%; and a BLAST *e*-value < 1e − 50. The virtual taxa (VT) table was constructed from randomly resampled AM sequences (830 sequences/sample) resulting in 134 VTs (Table S2). Due to uneven sequencing depth, two samples of *A. nepalensis* and two samples of *S. wallichii* did not meet the required amount of AM sequences for random sampling and thus were excluded from the analysis (Table S1).

For the total fungi (including ECM fungi) (2,649,678 reads), pair-end reads were merged using fastq-join (Aronesty 2013). The ITS2 region was extracted using ITSx (v1.0.11) (Bengtsson-Palme et al. 2013) before processing. Chimeric sequences were removed using VSEARCH (v2.4.3) (Rognes et al. 2016), and sequences at the 97% similarity level were clustered using UPARSE, which was implemented in USEARCH (v8.1.1861) (Edgar 2013). Subsequently, the most abundant sequences per OTU were obtained using MAFFT (v7.222) (Katoh et al. 2009) and subjected to identification against the UNITE + INSD dataset (accessed in February 2022) (Abarenkov et al. 2020) using BLASTn (at the species level, sequence similarity ≥ 97%; at the genus level, sequence similarity ≥ 80%). The OTU table was constructed from randomly resampled sequences (2000 sequences/samples) resulting in 2861 OTUs including singletons. Due to uneven sequencing depth, 11 samples of *A. nepalensis* and 10 samples of *S. wallichii* did not meet the required amount of fungal sequences and were excluded from the analysis (Table S1). In total, 34 *A. nepalensis* trees (11 SA, 12 LA, 11 RF) and 20 *S. wallichii* trees (7 SA, 8 LA, 5 RF) were analyzed. Only OTUs with an abundance of ≥ 0.1% were considered for analysis (Table S4).

The ECM fungal diversity and community analysis was done only for *A. nepalensis*. For this, the ECM fungal OTUs were first manually sorted from total fungal OTUs following FUNGuild (Nguyen et al. 2016). Only ECM fungal OTUs with the confidence of high probable or probable to be ECM were used. Due to the low number of ECM sequences per sample, the ECM sequences were randomly resampled for 490 sequences/samples (13 samples), remaining 8 samples with low ECM sequence abundance were resampled for 100 sequences/samples and then all converted to percentage values to prepare the OTU table (Table S4) containing 31 OTUs. In total, 21 *A. nepalensis* trees were analyzed (5 SA, 11 LA, 5 RF) (Table S1).

### Statistical analyses

The rarefaction curve (Fig. S1) for root composite sample was prepared using the package *mobr* (McGlinn et al. 2021) in the R statistical program (R Core Team 2019). Diversity estimates (observed richness, Shannon index, InvSimpson index) were calculated using the R packages *vegan* (Oksanen et al. 2022) and *OTUtable* (Linz et al. 2017). Differences in the diversity indices and the relative abundance of selected taxa among land use and tree species were analyzed by two-way ANOVA (with type III correction) and post hoc Tukey HSD test using R package *Stats*. In the case of non-normal data even after transformation, the pairwise Wilcoxon test was done. For community analysis Canoco (v5.00) software was used (ter Braak and Šmilauer 2012). The abundance of AM fungal VTs and ECM fungal OTUs were centered and standardized by Hellinger transformation before partial redundancy analysis (RDA). The total fungal dataset was log-transformed before partial canonical correspondence analysis (CCA). Latitude, longitude, and elevation of each sampling site were taken as covariates. The effects of land use, tree species, tree dbh, and site properties (slope, aspects, and soil types) were tested by the global permutation test of partial RDA and CCA. Pairwise comparisons of AM, ECM, and total fungal community composition between SA-LA, SA-RF, and LA-RF were done using the package *BiodiversityR* (Kindt and Coe 2005) and the indicator species analysis was done using the package *indicspecies* (Cáceres and Legendre 2009). Due to a low number of ECM morphotypes per *Alnus* tree, ECM morphotypes were not statistically analyzed.

## Results

### AM fungal diversity and community composition

Altogether, 134 VTs were included in the AM dataset (Table S2). The two-way ANOVA showed that there was no difference in AM diversity between trees (Table S9). Land use affected diversity indices only in *S. wallichii*, but there was no clear trend (Table 2).

**Table 2:** Diversity estimates of AM, ECM, and total fungi (mean ± SD)

**Taxa**	**Tree species**	**Land use**	**Observed richness**	**Inv_Simpson**	**Shannon**
**AM fungi**	*A. nepalensis*	SA	29.6 ± 5.6(a)	3.9 ± 1.4(ab)	1.8 ± 0.3(a)
		LA	25.3 ± 6.0(ab)	5.2 ± 2.7(ab)	1.8 ± 0.5(a)
		RF	22.6 ± 5.6(ab)	4.5 ± 2.6(ab)	1.6 ± 0.5(ab)
	*S. wallichii*	SA	20.9 ± 12.2(bc)	3.1 ± 0.2(a)	1.4 ± 0.6(ab)
		LA	23.0 ± 6.3(abc)	6.3 ± 0.4(b)	2.1 ± 0.3(a)
		RF	14.2 ± 3.3(c)	2.6 ± 0.5(a)	1.1 ± 0.4(b)
**ECM fungi**	*A. nepalensis*	SA	4.8 ± 2.4(a)	1.4 ± 0.4(a)	0.4 ± 0.4(a)
		LA	2.6 ± 1.1(a)	1.4 ± 0.4(a)	0.3 ± 0.3(a)
		RF	3.0 ± 2.0(a)	1.2 ± 0.4(a)	0.2 ± 0.3(a)
**Total fungi**	*A. nepalensis*	SA	100.4 ± 45.0(a)	4.6 ± 1.5(a)	2.1 ± 0.5(a)
		LA	105.2 ± 65.0(a)	4.9 ± 3.7(a)	2.1 ± 0.9(a)
		RF	158.0 ± 53.6(a)	8.8 ± 5.1(a)	2.7 ± 0.8(a)
	*S. wallichii*	SA	143.0 ± 34.9(a)	7.5 ± 2.9(a)	2.8 ± 0.4(a)
		LA	129.0 ± 39.3(a)	7.8 ± 4.9(a)	2.7 ± 0.5(a)
		RF	110.0 ± 72.1(a)	10.4 ± 3.7(a)	3.1 ± 0.5(a)

Unlike letters indicate significant differences as detected with the Tukey HSD test (*P* < 0.05)

*SA* short-term abandoned land, *LA* long-term abandoned land, *RF* regenerated forest

**Fig. 2:**
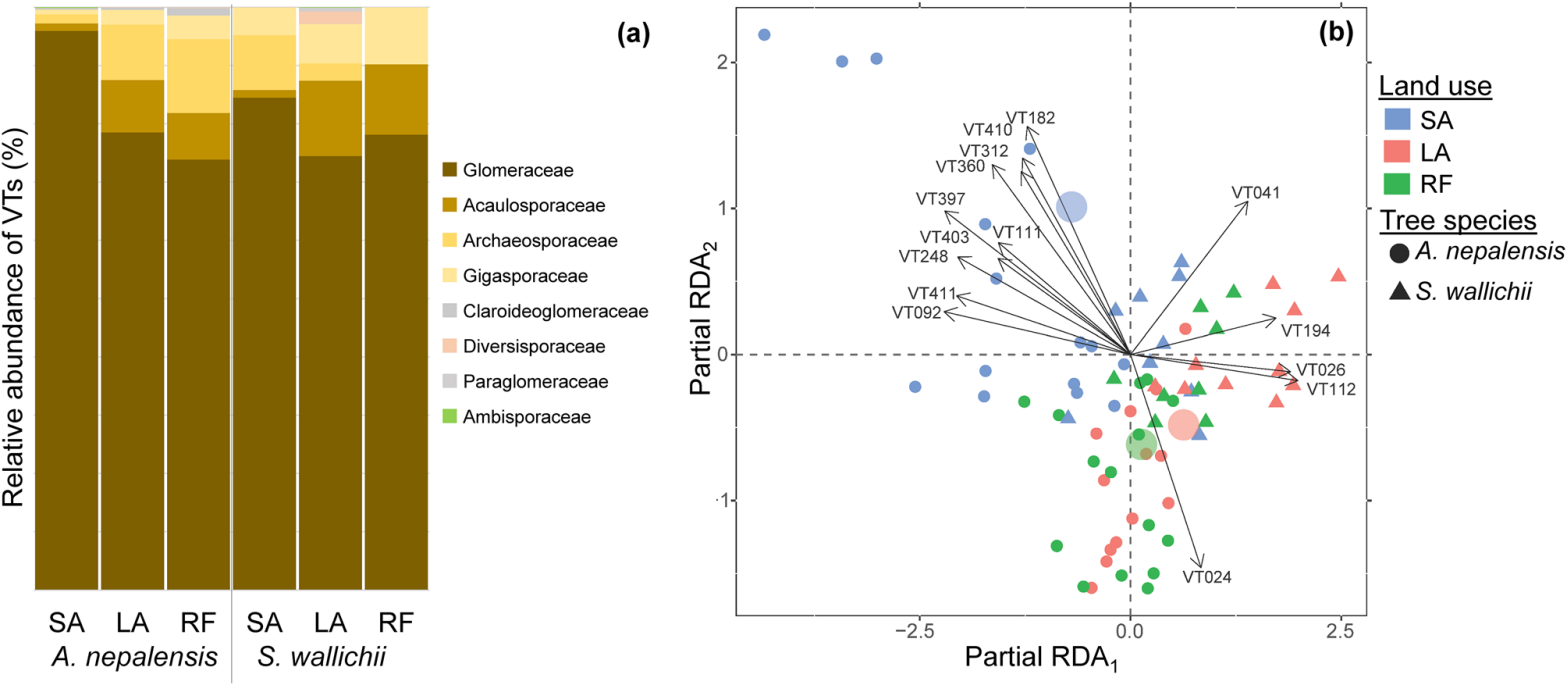
**a** Relative abundance (%) of VTs (at the family level) of AM fungi in *A. nepalensis* and *S. wallichii* among different types of land use. **b** VTs composition of AM fungi (variation explained by partial RDA_1_ = 3.19% and RDA_2_ = 2.82%) among different types of land use. Only 30 most dominant VTs are shown. Taxonomic information of VTs is listed in Table S2. Relative abundance (%) of VTs (at the family level) of individual trees is in Table S3. SA short-term abandoned land, LA long-term abandoned land, RF regenerated forest

Among the identified VTs, the majority were assigned to Glomeraceae (81%), followed by Acaulosporaceae (6%), Archaeosporaceae (6%), and Gigasporaceae (4%). Interestingly, the relative abundance of Archaeosporaceae in *A. nepalensis* and *S. wallichii* among land use types showed a different trend (Fig. 2a). *A. nepalensis* showed a higher relative abundance of Archaeosporaceae in LA than in SA (*P* < 0.05). In contrast, *S. wallichii* showed a higher relative abundance of Archaeosporaceae in SA than in LA and RF (*P* < 0.05). Additionally, land use type affects the relative abundance of Acaulosporaceae, and there was a significantly lower relative abundance in SA than other land use types (*P* < 0.05) in both trees.

Ordination analysis showed that the AM fungal community composition differed significantly among land use and tree species (Fig. 2b). Land use and tree species explained 4.5% (pseudo-*F* = 1.5, *P* < 0.05) and 2.9% (pseudo-*F* = 2.0, *P* < 0.05) variation in the VTs composition, respectively. Land use and tree species together explained 11.1% (pseudo-*F* = 1.6, *P* < 0.05) of the variation in the VTs composition. In a separate analysis of the land use change effect on *A. nepalensis* and *S. wallichii*, land use affected significantly only VTs composition of *A. nepalensis* (explained variation = 7.4%, pseudo-*F* = 1.5, *P* < 0.05). While testing the effect of site properties and tree dbh, only site slope (explained variation = 2.5%, pseudo-*F* = 1.7, *P* < 0.05) significantly affected AM fungal community composition (Table S10). Pairwise comparison of AM fungal community between land use types showed significant differences in SA-LA (*P* < 0.05), SA-RF (*P* < 0.05), and LA-RF (*P* < 0.05).

Some of the taxa were also exclusive to *A. nepalensis*, e.g., *Acaulospora* sp. (VT020), *Archaeospora* sp. (VT009), *Diversispora* sp. (VT054), *Glomus* spp. (VT064, VT065, VT073 etc.), *Paraglomus occultum* (VT238), and *Scutellospora nodosa* (VT261). For *S. wallichii*, examples of exclusive taxa included *Acaulospora* sp. (VT015), *Archaeospora* sp. (VT338), *Glomus intraradices* (VT100), *Glomus* spp. (VT068, VT087, VT142 etc.), *Ambispora* sp. (VT238), and *Claroideoglomus* spp. (VT056, VT358). Indicator species analysis showed that *Glomus* spp. (VT126, VT182, VT248, VT410, VT411) typically occurred in SA, *Archaeospora* sp. (VT005) and *Gigaspora decipiens* (VT039) in LA, and *Glomus* sp. (VT368) in RF (Table S11).

### ECM fungal diversity and community composition

In contrast to our field observation of ECM fungal fruitbodies near *S. wallichii*, we did not observe ECM morphotypes on its root tips. Therefore, no further analysis was performed in the case of *S. wallichii*. Regarding *A. nepalensis*, 9 different ECM morphotypes were detected, and 6 of them were identified by Sanger sequencing: 3 morphotypes of *Tomentella* spp., and one morphotype of *Inocybe* sp., *Cortinarius* sp., and *Sebacina* sp. (Table S8). Three other ECM morphotypes remained unidentified due to unsuccessful DNA extraction or contamination by other fungi (Table S8). Thirty-one ECM fungal OTUs, including genetically identical with 4 identified ECM morphotypes, were detected based on Illumina sequencing (Table S4). The diversity analysis of ECM fungal OTUs did not show any difference among land use types (Table 2).

**Fig. 3:**
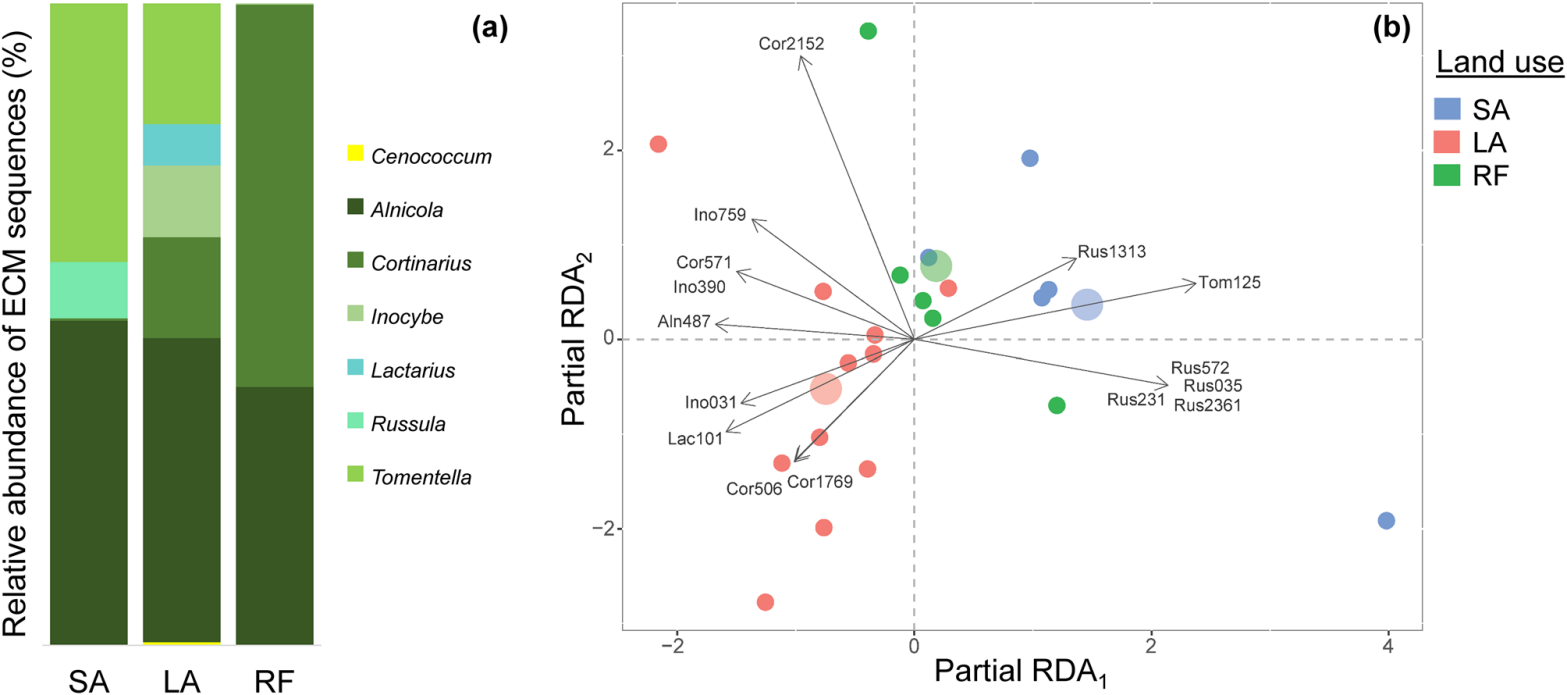
**a** Relative abundance (%) of ECM fungi (at the genus level) in *A. nepalensis* among different types of land use. **b** Community composition of ECM fungi (variation explained by partial RDA_1_ = 8.12% and RDA_2_ = 4.28%) among different types of land use. Only 15 most abundant ECM taxa are shown (abbreviation: Aln487 *Alnicola* sp. 487, Cor506 *Cortinarius* sp. 506, Cor571 *Cortinarius* sp. 571, Cor1769 *Cortinarius* sp. 1769, Cor2152 *Cortinarius* sp. 2152, Ino031 *Inocybe* sp. 031, Ino390 *Inocybe* sp. 390, Ino759 *Inocybe* sp. 759, Lac101 *Lactarius* sp. 101, Rus035 *Russula* sp. 035, Rus231 *Russula* sp. 231, Rus572 *Russula* sp. 572, Rus1313 *Russula* sp. 1313, Rus2361 *Russula* sp. 2361, Tom125 *Tomentella* sp. 125). Relative abundance (%) of ECM fungi (at the genus level) of individual tree is shown in Table S5. SA short-term abandoned land, LA long-term abandoned land, RF regenerated forest

Except for *Cenococcum* sp., all identified ECM fungal OTUs belonged to Basidiomycota. Within Basidiomycota, Agaricales was the most abundant, followed by Thelephorales and Russulales (Fig. 3a, Table S4). Taxa such as *Alnicola*, *Cortinarius*, and *Tomentella* were relatively abundant ECM fungal OTUs. The relative abundance of *Alnicola* followed the order SA > LA > RF, whereas *Cortinarius* showed the opposite trend RF > LA > SA. *Inocybe* showed a relative abundance in the order of LA > RF and *Tomentella* SA > LA. The taxa *Cenococcum* and *Lactarius* were found only in LA, and *Russula* was found only in SA. However, no significant differences were found in the relative abundance of these taxa among land use types except for *Cortinarius* (RF > SA (*P* < 0.05)).

Ordination analysis showed that the composition of the ECM fungal community of *A. nepalensis* did not differ significantly among land use types (Fig. 3b). Likewise, tree dbh and site properties (slope, aspect, and soil types) did not affect ECM fungal community composition significantly among land use types (Table S10). Pairwise comparison between land use types showed no significant difference in ECM fungal community composition. Based on the ordination diagram, ECM fungal taxa such as *Cortinarius* sp. 2152 and *Inocybe* sp. 759 are relatively more abundant in RF; likewise *Cortinarius* sp. 506, *Cortinarius* sp. 1796, *Inocybe* sp. 31, *Lactarius* sp. 101 in LA, and *Tomentella* sp. 125, including other *Russula* OTUs in SA. Indicator species analysis did not detect particular ECM fungi that are typical for SA, LA, or RF.

### Diversity and community composition of root-associated total fungi

**Fig. 4:**
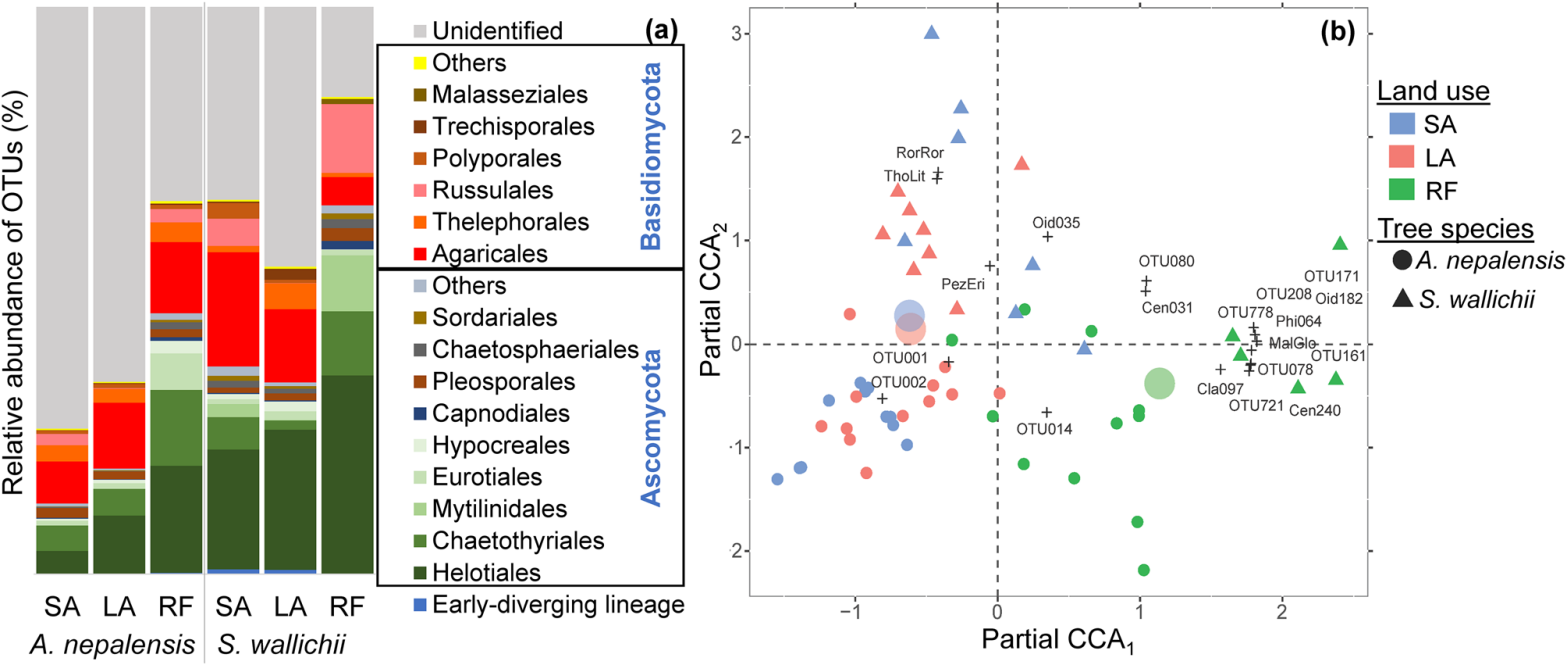
**a** Relative abundance (%) of total fungi (at the order level) in *A. nepalensis* and *S. wallichii* among different types of land use. **b** Community composition of total fungi (variation explained by partial CCA_1_ = 4.8% and CCA_2_ = 3.25%) among different types of land use. Only the 20 most abundant OTUs are shown (abbreviation: MalGlo *Malassezia globosa*, PezEri *Pezicula ericae*, RorRor *Roridomyces roridus*, ThoLit *Thozetella lithocarpi*, Cen031 *Cenococcum* sp. 031, Cen240 *Cenococcum* sp. 240, Cla097 *Cladophialophora* sp. 097, Oid035 *Oidiodendron* sp. 035, Oid182 *Oidiodendron* sp. 182, Phi064 *Phialocephala* sp. 064 and unidentified OTUs are abbreviated as OTU followed by its ID, e.g., OTU001, OTU002 (Table S4)). Relative abundance (%) of total fungi (at the order level) of individual trees is in Table S7. SA short-term abandoned land, LA long-term abandoned land, RF regenerated forest

Together, 2861 OTUs were present in the rarefied total fungal dataset. There were no significant differences in diversity indices among land use and tree species (Table 2). The total fungal dataset contained 65% of Ascomycota, 12% of Basidiomycota, and 4.5% of the early-diverging lineages (Fig. 4a). Approximately 17% of the fungal OTUs were unidentified. The land use type affected the relative abundance of Ascomycota (*P* < 0.05) (Table S9). In case of *A. nepalensis*, the relative abundance of Ascomycota was higher in RF and LA than in SA (*P* < 0.05 and *P* < 0.05, respectively); in *S. wallichii*, no significant differences were found. Helotiales and Chaetothyriales were relatively abundant taxa of Ascomycota. Land use and tree species both significantly affected the relative abundance of Helotiales (*P* < 0.05 and *P* < 0.05, respectively). The relative abundance of Chaetothyriales was affected only by the interaction effect of land use and tree species (*P* < 0.05). Agaricales and Thelephorales were relatively abundant taxa of Basidiomycota.

Ordination analysis showed that the total fungal community composition differed significantly among land use types that explained 6.8% (pseudo-*F* = 1.7, *P* < 0.05) of the variation (Fig. 4b). Likewise, tree species explained 3.6% (pseudo-*F* = 2.0, *P* < 0.05) of the variation in the total fungal community composition. Both abandoned land (SA and LA) are clearly separated from RF in the ordination diagram. While testing the effect of tree dbh and site properties, slope (explained variation = 2.4%, pseudo-*F* = 1.2, *P* < 0.05) and soil types (explained variation = 2.4%, pseudo-*F* = 1.2, *P* < 0.05) were found to affect total fungal community composition significantly (Table S10). Pairwise comparison of total fungal community between each land use type test showed significant differences between SA-LA (*P* < 0.05), SA-RF (*P* < 0.05), and LA-RF (*P* < 0.05).

Based on the ordination diagram, taxa such as OTU171, OTU462, and *Oidiodendron* sp. 182 are relatively more abundant in RF, whereas taxa such as OTU001, OTU002, OTU004, and *Pezicula ericae* in SA and LA. Indicator species analysis showed that taxa such as *Mycena* sp. 013, *Coniothyrium platani*, and three other unidentified OTUs (OTU002, OTU007, OTU039) are typical for SA, and unidentified OTUs (e.g., OTU017, OTU033, OTU082) for LA and *Cyphellophora* sp. 056, *Cladophialophora* sp. 009, *Phialocephala* sp. 064 etc. for RF (Table S11).

## Discussion

### AM community

Our results show that there was barely any influence of land use type associated with decreasing disturbance (SA > LA > RF) on the AM fungal diversity associated with the studied trees (Table 2). This is in agreement with our previous findings based on a soil fungal analysis (Balami et al. 2021). We also observed a dominance of Glomeraceae in all land use and tree species (Fig. 2a), which is a common feature noted in various previous studies (Põlme et al. 2016; Zhao et al. 2018). In contrast to our second hypothesis, the AM community of RF is more similar to LA than SA, which could indicate its fast recovery or transformation (Fig. 2b). In addition to decreasing disturbance pressure, the reason could also be a decreasing abundance of forbs and grasses, which are important AM hosts (Wang and Qiu 2006). In both trees, SA showed a significantly lower relative abundance of Acaulosporaceae than the other land use types. This could be due to a higher disturbance in SA because Acaulosporaceae have very delicate and diffuse hyphae which are sensitive to disturbance (van der Heyde et al. 2017).

Consistent with our third hypothesis, land use had a significant effect on AM species composition, which was more pronounced than the effect of tree identity. Although AM fungi are considered to have low host specificity (Martin 2016), an effect of host identity, as indicated by Šmilauer et al. (2020), Sepp et al. (2019), and Davison et al. (2011), was also observed. The relative abundance of Archaeosporaceae followed a different trend between trees (LA > SA in *A. nepalensis* but SA > LA in *S. wallichii*). In particular, *Archaeospora* sp. (VT004 and VT005) showed this trend. This means that some species of *Archaeospora* respond differently to land use depending on their host. We also found a significant difference in the overall AM species composition between tree species. The absence of a significant effect of land use on the AM community of *S. wallichii* in the separate analysis is probably caused by the lower number of samples compared to *A. nepalensis*. We found AM taxa such as *Acaulospora*, *Archaeospora*, *Diversispora*, *Glomus*, *Paraglomus*, and *Scutellospora* in *A. nepalensis* and *S. wallichii* which were previously recorded by Pandey et al. (2016) from Northeast India. On the other hand, *Claroideoglomus*, *Redeckera*, *Racocetra*, and *Gigaspora* were found to be new AM taxa associated with *A. nepalensis*.

In general, we found that land use had an effect on AM symbionts of the tree species studied. However, the consequences of this finding are difficult to assess without knowledge of the traits of individual VTs and their relationships with different host species.

### ECM community

We did not find any significant differences in diversity (Table 2), taxonomic, and species composition of ECM fungi between land use types (Fig. 3). This could be attributed to the high variability of ECM communities caused by patchy species occurrence (Koide et al. 2005; Tedersoo et al. 2009). However, some differences are notable. The relative abundance of *Cortinarius* sequences is significantly higher in RF than in SA, which could be related to the ability of *Cortinarius* species to grow in recalcitrant organic substrates available in the forest due to its class II peroxidase enzymes (Bödeker et al. 2014). Similarly, Correia et al. (2021) reported a high abundance of *Cortinarius* in long-established beech forests. Additionally, *Cortinarius* differed from most other detected genera by extensive extraradical mycelia (= medium-fringe exploration type), which could facilitate nutrient uptake in mature forests (Hobbie and Agerer 2010). By contrast, the relative abundance of *Alnicola* sequences gradually decreases toward natural stages. Although we were unable to identify *Alnicola* from ECM morphotypes by Sanger sequencing, we found its fruitbodies in all three land use types. However, it is known that fruitbody abundance not always corresponds to the abundance of ectomycorrhizae (Dahlberg et al. 1997). The relative abundance of *Tomentella* spp. decreasing with time since abandonment (SA > LA) could be related to their ability to establish shortly after disturbance (e.g., fire) from the spore bank (Baar et al. 1999) because of their thick-walled spores. Correia et al. (2021) similarly reported that *Tomentella* prefers recent forests to long-established ones. Although *Russula* is traditionally a late-stage ECM, we found *Russula* in SA (early-stage). However, fine-scale studies also contradict the argument of *Russula* being a late-stage ECM (Liang et al. 2004; Bergemann et al. 2006). Moreover, *Russula* is such a species-rich genus (Sarnari 1998) that it is difficult to generalize its ecology.

The imbalance between number of ECM morphotypes and OTUs is probably due to the impossibility of capturing and recognizing very rare ECM morphotypes. The absence of the *Alnicola* ECM morphotype is very interesting. Perhaps it forms mycorrhizae deeper than we were able to reach or its DNA was particularly sensitive to degradation. Five of the ECM genera found in our study had already been reported by Põlme et al. (2013) in *A. nepalensis* from Yunnan, China. Fruitbodies of *Russula* have also been reported from an *A. nepalensis* forest in Nepal (Adhikari 2000). The relatively low number of ECM taxa is in agreement with previous observations of *Alnus* spp. (Tedersoo et al. 2009).

### Total fungal community

Similar to the results of AM and ECM fungal diversity, there was no effect of land use on the total fungal diversity. Approximately 17% of the total fungal OTUs remain unidentified to order level, which highlights the importance of basic taxonomic research. At the higher taxonomic level, we found differences in the relative abundance of Ascomycota and one of its orders, Helotiales, between land use types. However, it is difficult to reach any conclusion from higher taxonomic levels because they include genera with diverse ecological demands. The identified OTUs of Helotiales include fungi such as *Pezoloma* sp., *Cladophialophora* sp., *Pezicula ericae*, *P*. *rhizophila*, *Phialocephala* sp., *Rhizodermea veluwensis*, and *Lachnum* sp. that are either saprotrophs or root endophytes and *Oidiodendron* spp. and *Meliniomyces* sp. that are either saprotrophs or root endophytes or ericoid mycorrhizal fungi (Põlme et al. 2020). As we expected, the main difference in species composition was found between successional stages (SA + LA) and RF, probably because of the long-term undisturbed conditions which favored fungi associated with ericoid trees and shrubs.

In contrast to our hypothesis, the effect of host tree on species composition was less pronounced than was land use change. This means that tree species specialists (ECM fungi, specialized litter decomposers) only created a minor part of the detected community. The resulting fungal assemblage was mainly composed of non-host-specialized ericoid fungi and root endophytes and litter decomposers from the surrounding vegetation.

## Conclusion

Succession of abandoned land affected root fungi associated with *Alnus nepalensis* and *Schima wallichii* in a different way depending on studied parameters and functional groups of fungi. Although neither of the studied groups showed a clear effect of land use on its diversity, their species composition clearly differed. The difference of AM fungal communities between SA and other land use types indicates a rather fast transformation toward communities of mature forests and highlights the potential of AM-associated indigenous trees to spontaneously restore previously intensively exploited areas. On the other hand, succession of total fungal communities proceeds much more slowly, possibly due to their incomparably greater diversity and composition of species specialized to a variety of environments and substrates. Although a significant effect of land use on the ECM fungal composition associated with *A. nepalensis* was not found, some preferences of *Tomentella*, *Alnicola*, and *Russula* for young stages and *Cortinarius* for regenerated forests were identified. The presence of both mycorrhizal types could give *A. nepalensis* the ability to take advantage of both the rapidly changing AM community and the slow succession of ECM symbionts. The effect of tree identity on AM and total fungal communities was found to be less pronounced than that of land use, which points to the importance of surrounding vegetation and the occurrence of generalist fungi not strictly associated with the studied tree species.

### Supplementary Information

Below is the link to the electronic supplementary material.

Supplementary file1 (XLSX 2004 KB)

Supplementary file2 (TIF 31602 KB)

## Data Availability

The raw sequence reads have been deposited into the GenBank Sequence Read Archive (SRA) database under the accession numbers PRJNA851964 and PRJNA853617.
