# Supplementary figures and images for: How do root fungi of Alnus nepalensis and Schima wallichii recover during succession of abandoned land?
Source: Mycorrhiza. 2023 Sep 13;33(5-6):321–32. doi: 10.1007/s00572-023-01124-6 (PMC10752848; doi:10.1007/s00572-023-01124-6)

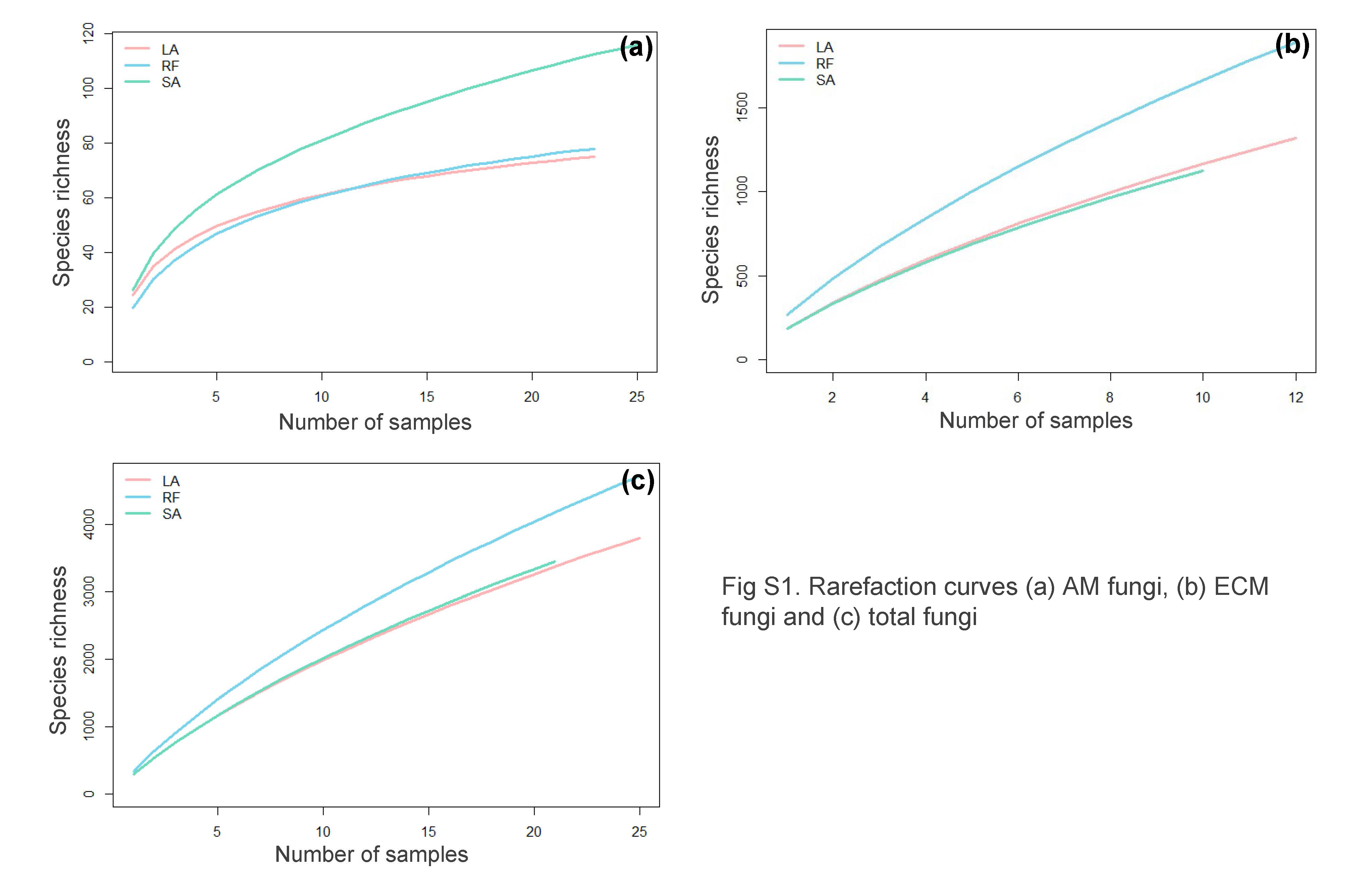

Supplement: Supplementary file 2 — Supplementary file2 (TIF 31602 KB) [file 572_2023_1124_MOESM2_ESM.tif]
